# Towards mastery of complex visuo-motor transformations

**DOI:** 10.3389/fnhum.2013.00032

**Published:** 2013-02-13

**Authors:** Herbert Heuer, Sandra Sülzenbrück

**Affiliations:** IfADo - Leibniz Research Centre for Working Environment and Human FactorsDortmund, Germany

**Keywords:** motor learning, internal model, transformation, tool use, explicit learning, implicit learning

## Abstract

In this paper we review and integrate a set of findings on learning the transformation of a sliding first-order lever, a type of tool with a prominent role in minimal access surgery. Its kinematic transformation is characterized by the so-called fulcrum effect, the inversion of the movement direction of the tip of the lever relative to that of the hand for rotations. A second characteristic is gain anisotropy, which results in curved paths of the tip of the lever for straight paths of the hand and vice versa. An internal model of the kinematic transformation is acquired during practice, the accuracy of which can be assessed in visual open-loop test trials. The accuracy of the acquired internal model is enhanced when visual closed-loop control during practice is impeded, and the accuracy of the internal model is reduced when closed-loop control during practice is facilitated. The internal model consists of a rapidly acquired line-symmetric approximation to the transformation of the sliding lever and a slowly acquired fine tuning. The fine tuning is local, that is, it is specific for the region of the workspace encountered during practice. The internal model is transferred to other regions of the workspace, but not adjusted to the fine tuning appropriate for these regions. Whereas the symmetry approximation is most likely explicit, the fine tuning seems to be represented implicitly. Findings on the straightness of the paths of the tip of the lever and the hand suggest that the internal model of the transformation is confined to initial and final positions of aimed movements, whereas their path is not strictly controlled, but affected by the dynamic transformation of the tool. Only when visual closed-loop control is possible, the path of the effective part of the tool is straightened. These characteristics of the internal model of the sliding first-order lever and its acquisition may be partly specific to sufficiently complex extrinsic transformations that arise from mechanical or electronic extensions of the body.

Movement execution involves a series of transformations (cf. Heuer and Massen, 2013). For example, efferent commands are transformed into muscular forces, muscular forces are transformed into joint torques, joint torques are transformed into joint rotations, joint rotations are transformed into movements of an end effector such as the hand. Planning and control of a voluntary movement of the end effector requires an internal model of the series of transformations, more precisely, an inverse model that allows to determine the input needed for a certain intended output (Heuer, 1983, p. 15; Wolpert and Kawato, 1998; Kawato, 1999). Such a model has to be plastic because the transformations are subject to change on different time scales (e.g., Körding et al., 2007). They change slowly in the course of bodily growth and involution, they change rapidly in the course of fatiguing exercises. Plasticity becomes possible because the brain has access not only to the neural input of the neuro-mechanical series of transformations, but also to the mechanical output and to intermediate mechanical variables by means of vision and proprioception.

The series of transformations, which is intrinsic to the body, is extended by extrinsic transformations when a tool is used. From an observer perspective, the difference between intrinsic and extrinsic transformations is fairly obvious, but from the perspective of the user of the tool the difference may be rather graded (cf. Heuer and Sülzenbrück, 2013a). On the one hand, movements with and without a tool may lead to similar perceptual experiences. For example, a classic observation, dating back at least to Descartes ([1637]1958), is the projection of tactile sensations into the outside world when we touch objects with a stick. Thus, for the haptic perception of the location of an object it does not make much of a difference whether we touch it with a finger or with a hand-held stick. Such phenomenological observations are complemented by physiological data. For example, Iriki et al. (1996) observed changing receptive fields of certain parietal neurons when a tool was used. On the other hand, a clear difference between ones own limbs and their extension by tools remains. The hand is not just replaced by a tool. It is evident that extrinsic transformations can change more rapidly and radically than intrinsic transformations. It is also likely that successful tool use can invoke higher cognitive processes such as mechanical reasoning in addition to basic processes of sensori-motor adaptation (Johnson-Frey, 2003).

In this paper we review and integrate a set of findings on learning a complex extrinsic transformation as it is inherent to a sliding first-order lever. At first glance this may appear as a rather esoteric kind of tool to study. However, this type of tool has a prominent role in minimal access surgery, which represents one of the currently greatest professional challenges of human sensori-motor skills (cf. Villegas et al., 2003). We start with a description of the transformation of the sliding lever. Following this, we present some findings which suggest a trade-off between visual closed-loop control during practice and the acquisition of an internal model of the extrinsic transformation. The main body of the paper will then deal with the characteristics of the internal model.

## The transformation of the sliding first-order lever

**Figure 1:**
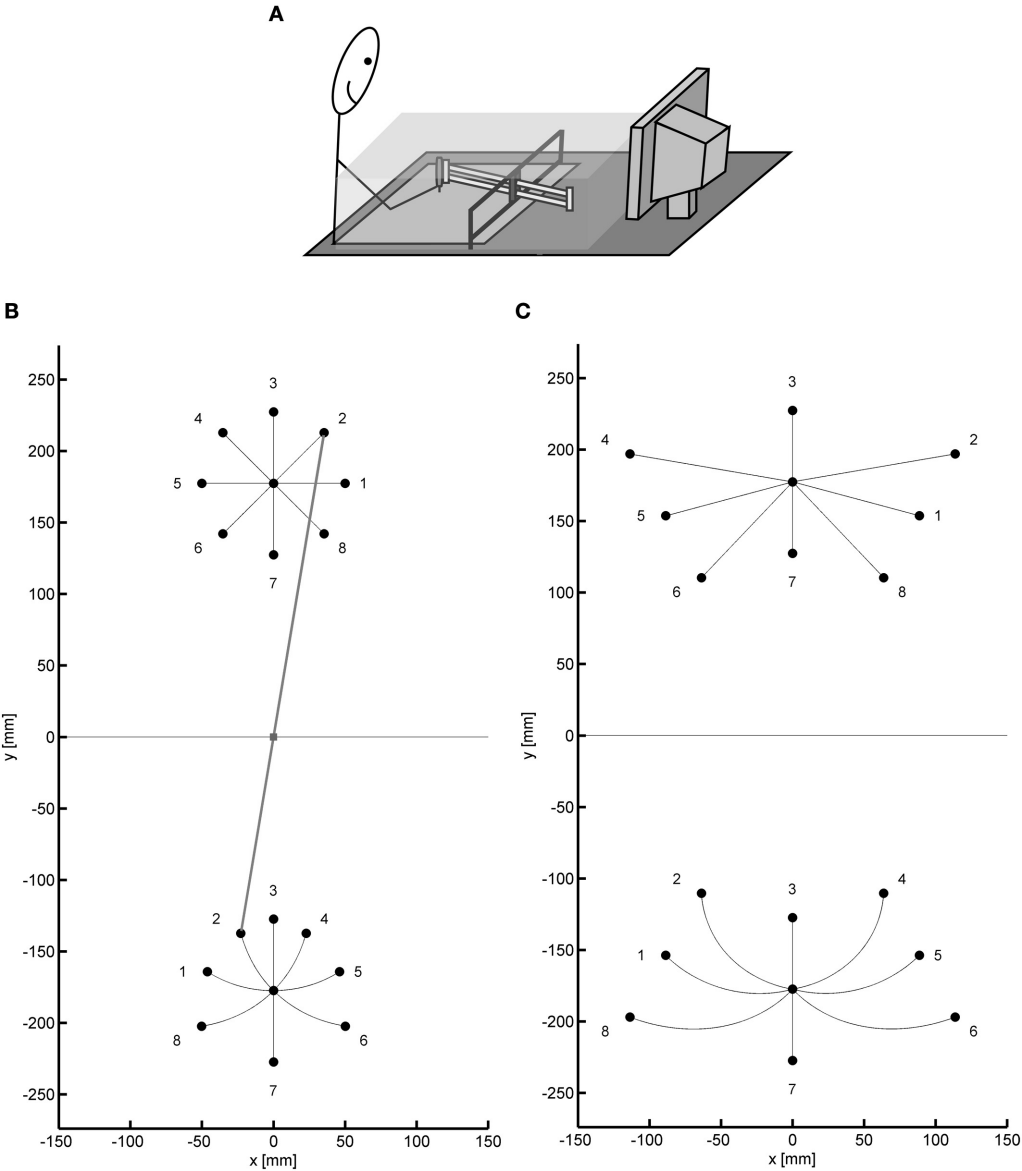
**(A)** Sketch of the typical experimental setup. **(B,C)** Two target configurations used in the series of experiments, shown with straight paths of the tip of the lever (and the cursor) and appropriately curved paths of the hand. Target positions for the cursor and corresponding positions for the hand are numbered 1–8. In **(B)** the position of the lever is displayed for the movement to target 2 (gray line).

Figure 1A shows the basic set-up of several experiments with the sliding first-order lever. The ball bearings of the lever were almost frictionless and allowed horizontal rotations around the fulcrum as well as translations, that is, forward and backward movements. Participants grasped a pen that was attached to the near end of the lever. Its position was recorded by means of a digitizer. The position of the tip of the lever was computed and presented on the monitor as the position of a cursor. The direct view of the hand and the lever was blocked by an opaque shield.

A tool such as the sliding lever implements both a kinematic and a dynamic transformation (see Heuer and Sülzenbrück, 2009, for a detailed description). The input of the kinematic transformation is the position of the hand, and its output is the position of the tip of the lever. The input of the dynamic transformation is the force exerted by the hand, and its output is the acceleration of the hand and thus the near end of the lever. The experimental setup allowed varying both transformations independently. The position of the cursor could indicate both the position of the tip of the lever (kinematic transformation of the sliding lever present) or of the pen (kinematic transformation absent). The pen could be attached to the physical lever (dynamic transformation of the sliding lever present) or it could be detached (dynamic transformation absent). When only the kinematic transformation is present, but not the dynamic one, we refer to the tool as a “virtual lever.”

The kinematic transformation of the sliding first-order lever can be described in different ways. It is mathematically quite simple when a Cartesian coordinate system with its origin in the fulcrum is chosen, as in Figures 1B,C:

$$\underline{c}=-\frac{l-\left|\underline{h}\right|}{\left|\underline{h}\right|}\times \underline{h}$$

with *c* as the position of the tip of the lever or, equivalently, of the cursor, (*x*_*c*_, *y*_*c*_), and *h* as the position of the hand, (*x*_*h*_, *y*_*h*_). The length of the lever is l, and |*h*| is the length of the effort arm. In terms of the movements produced, the kinematic transformation has two important characteristics. The more conspicuous one is the reversal of the direction of hand movements at the tip of the lever when the lever is rotated. This reversal is also known as the fulcrum effect (Gallagher et al., 1998). The less conspicuous characteristic is gain anisotropy, that is, the dependence of the visuo-motor gain on movement direction. For translations of the lever the gain is 1, that is, the amplitude of the tip of the lever is the same as that of the hand. For rotations, however, the gain varies. When the effort arm is longer than the load arm, the gain is less than 1, that is, the amplitude of the tip of the lever is smaller than that of the hand. When the effort arm is shorter than the load arm, the gain is larger than 1. When translations and rotations are combined to produce movements in various directions, the gain varies across directions. As a consequence of this gain anisotropy, straight movements of the hand will generally result in curved movements of the tip of the lever, and straight movements of the tip of the lever require particularly curved hand movements—a fact that makes certain surgical tasks quite difficult (e.g., Heuer et al., 2012).

The consequences of the kinematic transformation of the sliding lever for hand movements, which serve to produce straight movements of the tip of the lever, are illustrated in Figures 1B,C for two target configurations as they were used in our experiments. In both configurations there was a central start position. In the one configuration (Figure 1B) the targets were located on a circle around the start position (radius: 5 cm or similar) with angular separations of 45°. In the other configuration (Figure 1C) the targets were at the intersections of three concentric circles around the fulcrum with radii such as 12.75, 17.75, and 22.75 cm and three radial lines emanating from the fulcrum with angular separations such as 30°. From Figures 1B,C it is apparent that the kinematic transformation is quite complex when it is described in terms of directions and amplitudes, which are relevant parameters of motor control according to the vector-coding model (e.g., Vindras and Viviani, 1998). In fact, in particular with the target configuration of Figure 1C some participants tend to produce translations and rotations of the lever in sequence rather than concurrently (see right panel of Figure 5 in Heuer and Sülzenbrück, 2009), a strategy which simplifies the task in that for each translation or rotation the gain remains constant.

The dynamic transformation of the sliding lever plays only a minor role for the characteristics of movements with this tool. Acquisition of the internal model of the kinematic transformation is essentially unaffected by the presence or absence of the dynamic transformation (Sülzenbrück and Heuer, 2009a, 2010). In the absence of the kinematic transformation (when cursor movements represent movements of the pen at the proximal end of the lever), the dynamic transformation has almost no effects on movement characteristics (Heuer and Sülzenbrück, 2012a). Nevertheless, under certain conditions the dynamic transformation affects the curvature of the hand paths, as will be detailed below. This effect results from the inertial anisotropy of the sliding lever. For translations, the inertial resistance is constant, but for rotations it depends on the relative lengths of the effort arm and the load arm. A general consequence of the inertial anisotropy is a deviation of the direction of movement from the direction of force. If this is not taken into account during movement production, the paths of the hand will be curved. With the lever in our experiments, this curvature of hand paths was generally suited to reduce the curvature of the paths of the tip of the lever that results from the kinematic transformation. Thus, with respect to the curvature of the trajectories of the tip of the lever, the dynamic transformation tended to compensate the effects of the kinematic transformation.

## Closed-loop control and the acquisition of an internal model

Mastery of an extrinsic visuo-motor transformation requires its inversion, so that the hand movements that are appropriate for an intended movement of the effective part of the tool can be determined. In principle, the inversion can be achieved by open-loop control or by closed-loop control (Jordan, 1996). While in the former case a sufficiently accurate internal model of the transformation is required, in the latter case minimal or no adjustments of the parameters of the controller are sufficient.

In the control of limb movements, open-loop control and closed-loop control generally operate in parallel and combine their respective advantages (Cruse et al., 1990; Heuer and Massen, 2013). Thus, one could expect a trade-off between both mechanisms that invert the transformation. Obviously, when the internal model of the transformation is accurate, little is left for closed-loop control, but when the internal model is almost absent because of variable transformations or a transformation that is too complex to be learned, the load on closed-loop control is high. Perhaps less obviously, one might also expect a reverse influence during practice. Depending on the quality of closed-loop control, the performance benefits that accrue from the acquisition of an internal model vary. When closed-loop control is impeded, accurate performance depends on the acquisition of a sufficiently accurate internal representation. Therefore, performance benefits of learning such a representation are high, and a more accurate internal model of the transformation should be acquired. In contrast, when closed-loop control is facilitated, performance benefits of learning the representation are low, and the acquired internal model should be less accurate.

The evidence obtained with the sliding lever is consistent with the trade-off hypothesis. Sülzenbrück and Heuer (2011) compared the acquisition of the internal model of the kinematic transformation across three different practice conditions. In the first condition, visual feedback was presented concurrently during each movement. In the other two conditions visual feedback was terminal, that is, it was presented after the end of each movement only and could not be used for on-line corrections. The one kind of terminal feedback was knowledge of results—only the final position of the cursor was shown after the end of the movement. The other kind of terminal feedback was knowledge of performance—in this case the path of the cursor was shown in addition to its final position.

**Figure 2:**
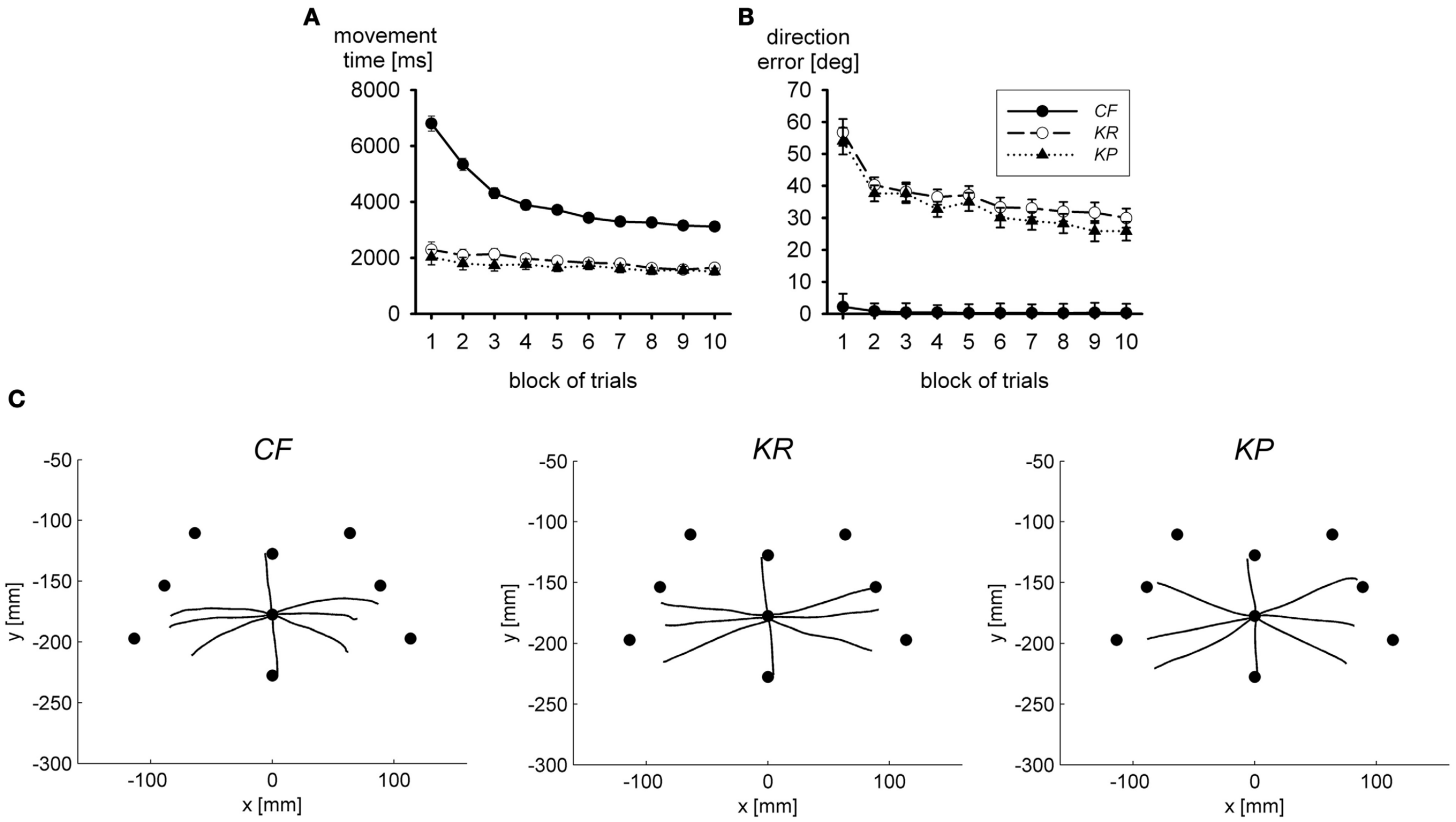
**Movement time (A) and direction error (B) during practice in three groups with different types of visual feedback; CF, continuous visual feedback; KR, knowledge of results (end position of the movement was shown together with the target); KP, knowledge of performance (path of the cursor and end position of the movement were shown together with the target).** In **(C)** averaged hand trajectories in a visual open-loop test are shown. Filled circles mark the correct end positions (after Sülzenbrück and Heuer, 2011).

Figure 2A presents movement time in the practice blocks of trials. With concurrent visual feedback, movement time was long and declined in the course of practice, whereas with terminal visual feedback movement time was much faster and essentially constant across practice blocks. The error of movement direction, shown in Figure 2B, exhibits the reverse pattern. It was large and declined in the course of practice with terminal visual feedback, but with concurrent visual feedback it was essentially 0 throughout practice. Thus, in terms of accuracy, there was no performance benefit of acquiring an internal model in the presence of concurrent visual feedback, but only in its absence. In terms of movement duration, there may have been some performance benefits; however, it is not clear to what extent the decline of movement time results from the acquisition of an internal model (and the thereby reduced load on closed-loop control) or from the optimization of the closed-loop controller.

The accuracy of internal models of extrinsic transformations can be assessed in visual open-loop tests in which the accuracy of performance critically depends on the accuracy of the model (Heuer, 1983, p. 46; Davidson et al., 2000). In Figure 2C the mean hand paths in such open-loop tests after practice with the different kinds of visual feedback are shown. Under these open-loop conditions movements were fairly inaccurate in all three groups, but after practice with concurrent visual feedback errors of direction were even stronger than after practice with terminal visual feedback. The same was true for the variable errors. Thus, by interfering with visual closed-loop control during practice one can facilitate the acquisition of the internal model of the visuo-motor transformation of the sliding first-order lever.

**Figure 3:**
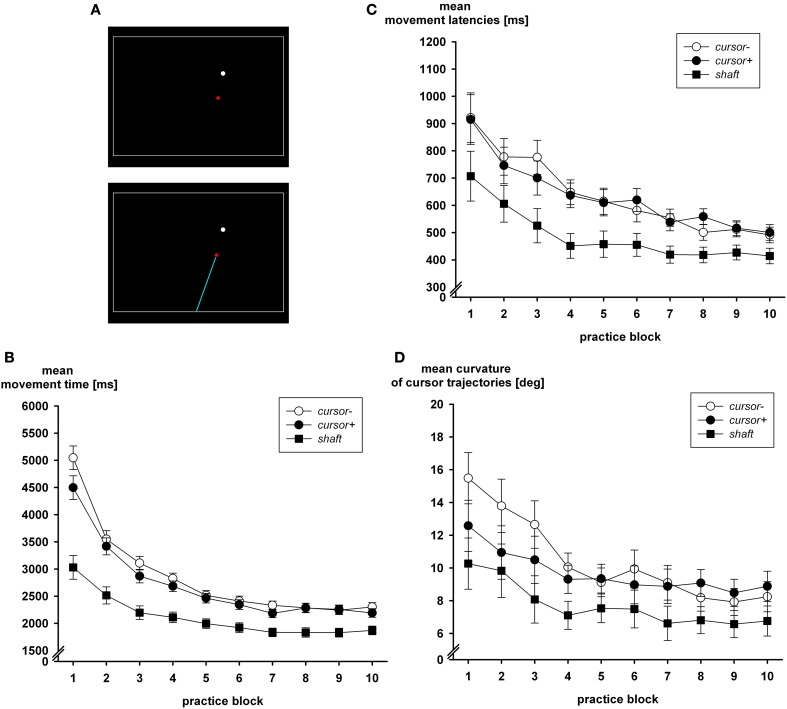
**(A)** Screenshots with only the cursor visible during practice (upper graph) and the additional visible shaft (lower graph), which emanated from a central position at the bottom of the monitor. Closed-loop performance in the three groups during practice blocks is captured by movement time **(B)**, latency **(C)**, and curvature **(D)** (after Sülzenbrück and Heuer, 2012).

In addition to the prevention of visual closed-loop control, its facilitation does also produce the expected effects, which in this case is a reduced accuracy of the acquired internal model of the extrinsic transformation. Wentink et al. (2002) observed faster performance in a simulated minimal access surgery task when the shaft of the laparoscopic instrument was presented on the monitor. With the visible shaft, the mechanical transparency of the tool is enhanced as compared to the task variant where only a cursor is visible (Heuer and Hegele, 2010; Sülzenbrück and Heuer, 2012, cf. Figure 3A). Figures 3B–D shows the effects of the visible shaft on visual closed-loop performance. In addition to the group who saw only the cursor (*cursor*−) and the group who saw the shaft of the instrument in addition (*shaft*), there was a group *cursor*+. In this third group only the cursor was visible as for group *cursor*−. However, group *cursor*+ received an initial explanation of the kinematic transformation of the sliding lever in the same way as group *shaft*, while this information was not given to participants of group *cursor*−.

From Figure 3B it is apparent that movement time was considerably faster with the visible shaft than when only the cursor was presented on the monitor. This difference was even larger in older adults than in young ones (Heuer and Hegele, 2010) as they participated in the study of Sülzenbrück and Heuer (2012). In addition the path of the cursor was straighter when the shaft was visible (Figure 3D), and movement latency—the time from presentation of the target to the start of the movement—was faster (Figure 3C). The difference in movement latency suggests that preparatory processes took less time when the shaft was visible, perhaps because of less involvement of open-loop control and the internal model of the visuo-motor transformation.

In a visual open-loop test, which followed the practice period, adaptive errors of direction were largest after practice with the visible shaft (28.0°) and smaller in groups *cursor*+ (12.6°) and *cursor*− (18.7°). Adaptive errors of direction are the direction errors of the hand in a visual open-loop test for which the presence of the transformation is instructed. From these errors those in a pre-test are subtracted in which the hand targets are presented and a 1:1 mapping of hand positions on cursor positions is instructed.

The findings reported thus far are consistent with the hypothesis of a trade-off between the quality of visual closed-loop control during practice and the accuracy of the acquired internal model of the visuo-motor transformation. From a practical perspective, the hypothesis suggests to impede closed-loop control during practice, e.g., by using terminal visual feedback, and to facilitate closed-loop control only after a fairly accurate internal model has been acquired. However, the situation becomes more complicated when additional findings are taken into account. In fact, overall the pattern of results obtained with different types of visual feedback during practice is fairly opaque.

For example, Heuer and Hegele (2010) used a virtual rather than a physical lever and observed benefits of the visible shaft for closed-loop control, but no clear effect on the accuracy of the acquired internal model. When one broadens the range of the visuo-motor transformations beyond that of the sliding lever, comparisons of the effects of concurrent and terminal feedback have sometimes found advantages of terminal feedback (e.g., Bernier et al., 2005; Heuer and Hegele, 2008a), but sometimes advantages of concurrent feedback (e.g., Peled and Karniel, 2012), and sometimes essentially no difference between conditions (Heuer and Hegele, 2008b). To this can be added observations on prism adaptation where terminal and concurrent visual feedback have been shown to result in different types of adaptive changes (e.g., Uhlarik and Canon, 1971). Similarly, Hinder et al. (2008, 2010) observed that concurrent and terminal visual feedback resulted in automatic recalibration and a cognitive strategy, respectively. On the other hand, Heuer and Hegele (2008a) could not find such a difference in tests of automatic after-effects and explicit knowledge. Thus, overall the differences between these different practice conditions are far from being clear. They are more a challenge for future research than a guideline for training schedules of minimal access surgery. The underlying mechanisms are not yet understood, but they are certainly more complex than suggested by the trade-off hypothesis which accounts for only a subset of the available findings (for a review of this line of research, see Sülzenbrück, 2012).

## Internal models of complex visuo-motor transformations

### Approximations and fine tuning

In studies of adaptation to extrinsic visuo-motor transformations, certain types of transformation are used preferably, namely visuo-motor rotations and—less frequently—gain changes. These transformations relate to the vector-coding hypothesis (Vindras and Viviani, 1998) according to which movement planning involves independent specifications of movement direction and amplitude. This notion has received considerable support both from behavioral data in humans (e.g., Favilla et al., 1989) and single-cell recordings in behaving monkeys (e.g., Georgopouplos et al., 1986). In addition, vector-coding allows a simple translation of a visually perceived target vector in the one plane, which points from start location to target location, into a movement vector in a different plane, which points from the current position of the hand to its target.

Adaptation to visuo-motor transformations can be conceived in terms of rotations and of length changes of the target vector to obtain the appropriately transformed movement vector. In fact, adaptation to rotations and gain changes differs both in behavioral characteristics and in neural substrates. Adaptation to changes of the visuo-motor gain is fairly rapid and generalizes across directions and amplitudes (Bock, 1992; Bock and Burghoff, 1997; Krakauer et al., 2000; Vindras and Viviani, 2002). In contrast, adaptation to visuo-motor rotations is slower and limited to a range of directions around the practiced one (Pine et al., 1996; Krakauer et al., 2000). Gain adaptation involves mainly subcortical structures (Krakauer et al., 2004), whereas rotation adaptation is accompanied by enhanced activity of cortical regions and the cerebellum (Ghilardi et al., 2000; Imamizu et al., 2000).

Turning to the visuo-motor transformation of the sliding first-order lever, it can be described in terms of rotations and gain changes, but this is a quite complex description which includes direction-dependent rotations and gain changes (cf. Heuer and Hegele, 2009). Even though adaptation to direction-dependent rotations and gain changes is possible (cf. Heuer and Hegele, 2008b; Hegele and Heuer, 2010a), these are not the ingredients of the internal model of the kinematic transformation of the sliding lever. The detailed analysis of the errors in visual open-loop tests after practice with the transformation of the sliding lever strongly suggests that the internal model captures the characteristics of the transformation in a different format.

**Figure 4:**
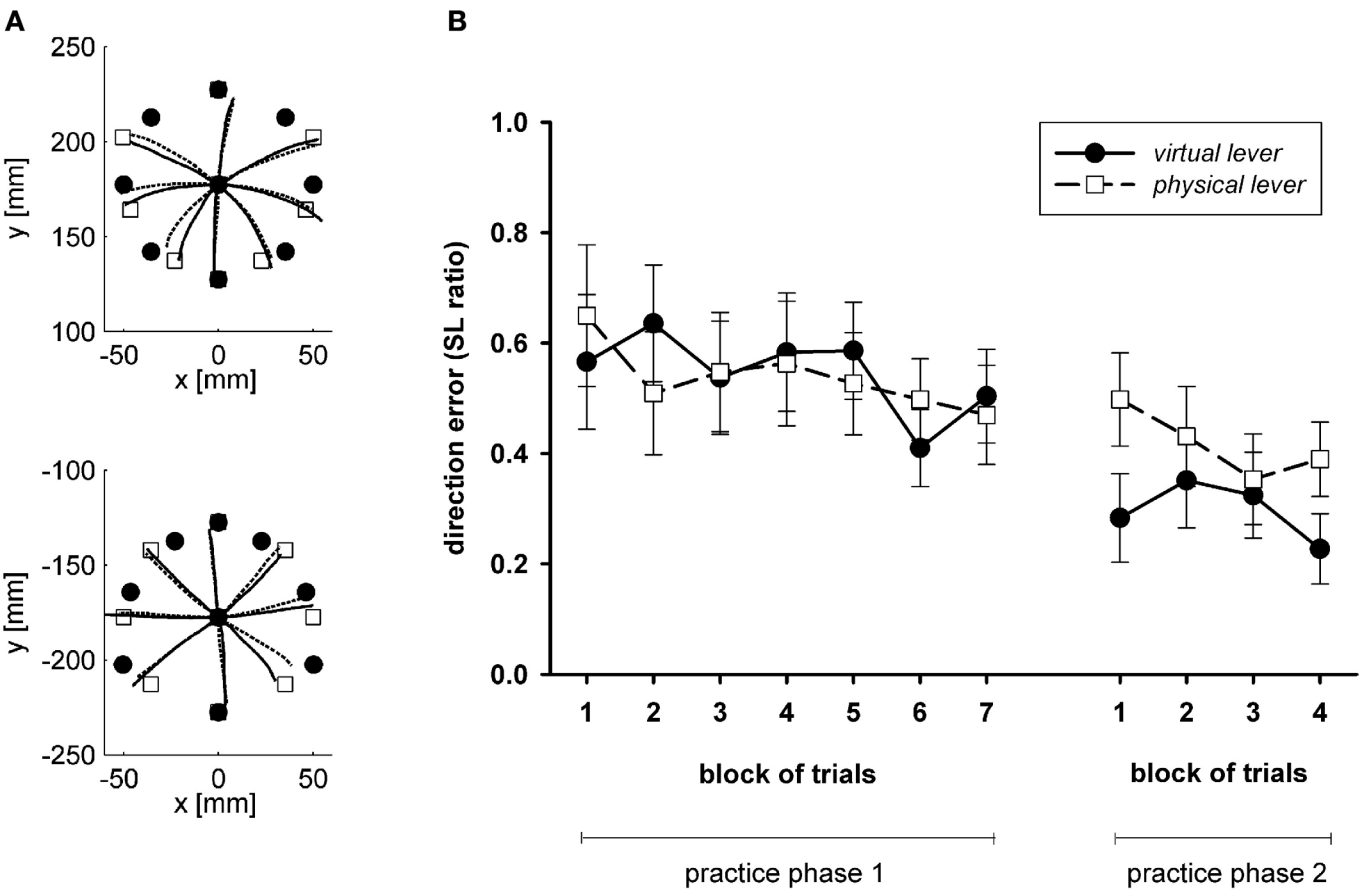
**(A)** Averaged trajectories of the cursor and the hand in an open-loop test after practice with the sliding first-order lever (after Sülzenbrück and Heuer, 2009a). **(B)** Mean ratios of the observed direction errors divided by the directional deviations of the symmetry approximation (SL ratios), with a value of 1 if the end position corresponds to the symmetry approximation, and of 0 if they correspond exactly to the correct target position (after Sülzenbrück and Heuer, 2010).

Figure 4A shows averaged trajectories of the cursor and the hand in an open-loop test after the end of practice, as reported by Sülzenbrück and Heuer (2009a). Movements do not end at their targets. However, the errors are highly systematic. Rather than at the targets, the movements end close to positions which are marked by outline squares. These are the correct final positions according to a line-symmetric approximation. Basically, to transform the target vector into an appropriate movement vector, it is reflected at a horizontal axis in the sagittal plane which runs through the start position of the hand (or a vertical axis through the start position of the cursor). In the experiment of Sülzenbrück and Heuer (2009a) the deviations from the line-symmetric approximation were only small. In a subsequent experiment (Sülzenbrück and Heuer, 2010) we used the target configuration of Figure 1C rather than the configuration of Figure 1B and terminal rather than concurrent visual feedback. Under these conditions the final positions of hand movements deviated more from the line-symmetric approximation and were gradually shifted toward the correct positions. Figure 4B shows the mean ratios of the observed direction errors divided by the directional deviations of the symmetry approximation. These ratios are 1 if the movements end exactly at the positions according to the approximation, and they are 0 if they end exactly at the correct target positions.

The observed errors in open-loop tests strongly suggest that the internal model of the kinematic transformation of a sliding first-order lever does not consist of direction-dependent rotations and gain changes, but of a rapidly acquired line-symmetric transformation and a slowly acquired fine tuning. To what extent the fine tuning is acquired at all depends on practice conditions. For reasons described above, a more precise fine tuning is acquired with terminal than with concurrent visual feedback, and perhaps also with target configurations for which the symmetry approximation results in larger errors than for target configurations for which the symmetry approximation is more accurate.

**Figure 5:**
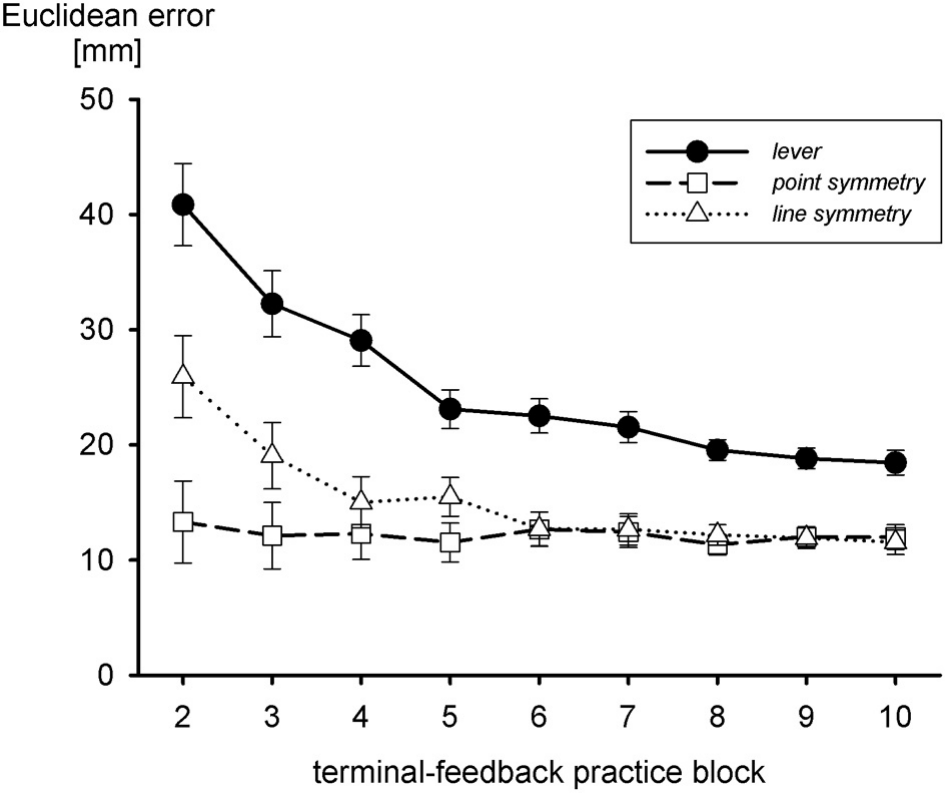
**Euclidean errors during practice with terminal visual feedback and three different transformations, the one of the sliding first-order lever, a line-symmetric one, and a point-symmetric one (after Heuer and Sülzenbrück, 2012c)**.

According to Werner and Bock (2010), an internal model of a line-symmetric transformation (either horizontal or vertical inversion) is acquired with an initial point-symmetric approximation (combined horizontal and vertical inversion), for which the hand movement is in the direction opposite to the target. Thus, acquisition of an internal model of the transformation of the sliding lever might also start with a point-symmetric approximation which precedes the line-symmetric one. The notion of a sequence of approximations to the internal model of the transformation of the sliding lever suggests that a line-symmetric transformation is acquired more rapidly and—to the extent that the fine tuning is incomplete—more accurately than the lever transformation. An internal model of a point-symmetric transformation should be acquired even faster. This is what Heuer and Sülzenbrück (2012c) observed, as shown in Figure 5. In fact, with the point-symmetric transformation accuracy of movements with terminal visual feedback was best from the very start and did not improve during practice. The analysis of movement endpoints during practice with the lever transformation showed the typical line-symmetric approximation, but only a very short-lived—if at all—point-symmetric approximation.

Thus far the symmetry approximation has been observed only with the lever transformation. It is not clear whether this approximation is also involved in the acquisition of internal models of other types of transformation. In addition, there are a number of questions that are not yet answered. A central question concerns the symmetry axis. In all experiments reported thus far, the start position of the hand, the tip of the lever, and the cursor were roughly aligned in the sagittal plane. The symmetry axis was also in this plane. What happens, however, when initially the lever is rotated, so that, for example, the initial hand position is to the left of the sagittal plane and the initial position of the tip of the lever (and of the cursor) to the right? Will the sagittal plane or the lever serve as the symmetry axis in such situations?

Our tentative answer to this question is: the lever. This answer is tentative because we have not yet run a dedicated experiment to identify the symmetry axes for a broader range of start-target configurations. Therefore, the answer is based on a re-analysis of the movements in the initial warm-up blocks of Experiment 1 of Heuer and Sülzenbrück (2009). In that experiment practice was with terminal visual feedback. The target configuration was of the type shown in Figure 1C, but in addition the configurations were rotated around the fulcrum so that in the start positions the lever was rotated clockwise or counter-clockwise relative to the sagittal plane. For the final positions of the hand in the left part of the workspace of the lever the mean Euclidean deviations from the correct positions were 24.6 mm, from the positions according to the line-symmetric approximation around the initial orientation of the lever 28.8 mm, and from the positions according to the line-symmetric approximation around a horizontal axis parallel to the sagittal plane 49.9 mm. For the final positions in the right part of the workspace the corresponding deviations were 31.4, 27.9, and 46.7 mm, respectively. Thus, the movements of the hand ended closer to the positions appropriate for a line-symmetric approximation around the axis defined by the initial orientation of the lever than to the positions appropriate for a line-symmetric approximation around an axis parallel to the sagittal plane.

### Local and global characteristics

The kinematic transformation of the sliding first-order lever is defined for its whole workspace. Thus, a rule that is acquired in some region of the workspace could be generalized to other regions. In contrast to studies of generalization, e.g., of adaptation to visuo-motor rotations (cf. Krakauer et al., 2000; Wang and Sainburg, 2005; Heuer and Hegele, 2011), generalization of the rule would imply different hand movements for same target vectors in different regions of the workspace. However, if indeed the internal model consists of the line-symmetric approximation and a fine tuning, generalization could take different formats. First, generalization could be restricted to the symmetry approximation. In this case the same hand movements would go along with same target vectors in different regions of the workspace. Second, fine tuning could generalize in addition. If the acquired fine tuning were local, it would remain invariant across different regions of the workspace. Again, same hand movements would go along with same target vectors. Third, if a general rule were learned for the fine tuning, generalization would be roughly appropriate for the particular fine tuning required in each region of the workspace. Only in this case same target vectors would be associated with different hand movements in different regions of the tool's workspace.

**Figure 6:**
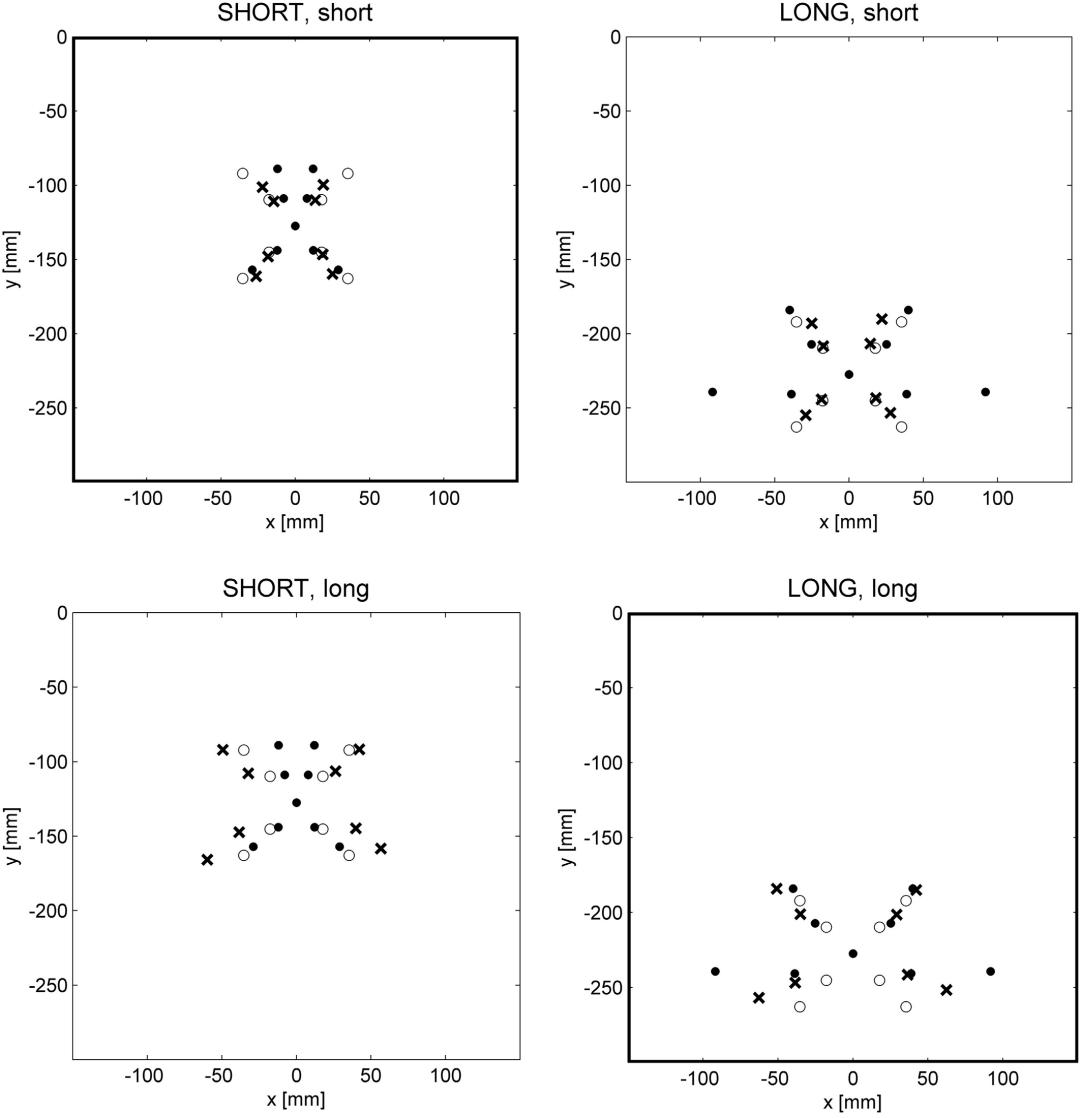
**Mean end positions of the hand (crosses) in two different regions of the workspace defined by a SHORT and a LONG effort arm of the lever (columns of graphs) after practice with a short and a long effort arm (rows of graphs).** Filled circles mark correct end positions of the hand, open circles those according to the line-symmetric approximation (after Heuer and Sülzenbrück, 2013b).

We studied the global vs. local characteristics of the internal model of the kinematic transformation of the sliding lever in a straightforward transfer experiment (Heuer and Sülzenbrück, 2013b). Participants practiced with target configurations of the type shown in Figure 1B. In three groups of participants, in the start positions the effort and the load arm were equally long, the load arm was longer, or the effort arm was longer. Thus, different groups of participants practiced in three different regions of the workspace of the lever. After practice, all participants were tested under visual open-loop conditions in the three regions, of which they had encountered only one during practice. Figure 6 shows the mean end positions of the hand together with the correct ones and the ones appropriate for the symmetry approximation for two groups of participants and two regions of the workspace in which transfer was tested.

From Figure 6 it is apparent that the required fine tuning, in particular that of movement directions, depends on the region of the workspace. For movements toward the participant (or downward on the monitor) the symmetry approximation is almost correct for the short effort arm, but for the long effort arm there are strong deviations; for movements away from the participant (or upward on the monitor) the symmetry approximation is almost correct for the long effort arm, and for the shorter effort arms the deviations become larger. The final positions of the hand movements of the participants deviate from the symmetry approximation toward the correct end positions. But these deviations are specific for the practice conditions and not for the tests, that is, the deviations from the symmetry approximation acquired during practice with a certain length of the effort arm are transferred to the tests with different lengths of the effort arm without taking the length of the effort arm into account. Accordingly the patterns of mean final positions are different across the two rows of graphs of Figure 6 (different regions of the workspace during practice), but not across the two columns (different regions of the workspace in transfer tests).

According to these findings, the fine tuning is represented locally, and the locally acquired fine tuning is generalized across the workspace of the lever together with the symmetry approximation. However, no general rule is acquired for the fine tuning that allows adjusting it to the different regions of the workspace. Such a rule, for example, could map the visuo-motor gain of rotations of the lever to the length of the effort arm which varied both within and between the target sets.

### Explicit and implicit representations

In general the execution of a movement involves early processes, such as an intention to reach for a certain object, which are subject to conscious awareness. In contrast, later processes, such as the changes of muscle lengths, remain outside conscious awareness. Somewhat intermediate processes, such as the adjustments of movements to extrinsic visuo-motor transformations, can be both (cf. Heuer et al., 2011; Heuer and Sülzenbrück, 2012b). In the present paper we have used the term “internal model” indiscriminately for implicit and explicit knowledge of the transformation, but it may be useful to more clearly distinguish the internal model which represents implicit knowledge and results in adaptation proper from explicit knowledge which is used for strategic corrections (cf. Heuer and Sülzenbrück, 2013a). For example, implicit knowledge of a visuo-motor transformation has to be acquired during physical practice, whereas explicit knowledge can also be instructed. Adjustments based on explicit and implicit knowledge are largely additive (Mazzoni and Krakauer, 2006; Sülzenbrück and Heuer, 2009b; Taylor and Ivry, 2011). However, interactions regarding the acquisition and use of the different types of knowledge can result when improved strategic corrections reduce the need to acquire implicit knowledge or when stronger implicit adaptive changes reduce the need for strategic corrections.

Implicit and explicit adjustments to visuo-motor transformations have a number of different characteristics in addition to the differences with respect to conscious awareness and the different ways of acquisition. Implicit adjustments to visuo-motor rotations are restricted to a range of target directions around the practiced ones, whereas explicit adjustments generalize across all target directions (Krakauer et al., 2000; Heuer and Hegele, 2008c); implicit adjustments are stable across the adult age range, whereas explicit adjustments decline (Bock, 2005; Heuer and Hegele, 2008c); implicit adjustments depend on intact cerebellar functions, whereas explicit adjustments do not (Taylor et al., 2010); different implicit adjustments cannot be acquired concurrently when the start position of the hand is the same, but different explicit adjustments can (Hegele and Heuer, 2010b); implicit adjustments to visuo-motor rotations are specific for a certain region of the workspace of the hand, whereas explicit adjustments generalize across a large range of the workspace (Heuer and Hegele, 2011). Most likely implicit and explicit adjustments are based on different types of error information (cf. Taylor and Ivry, 2012).

Explicit knowledge of the visuo-motor transformation of the sliding lever is clearly present. For its assessment we used a checkerboard pattern on the opaque shield that prevented direct sight of the hand and the lever. On this pattern the location of the start position was marked. In each trial the start position together with a target was presented on the monitor. Participants had to indicate the location of the near end of the lever appropriate for its distal end and thus the cursor to reach the target by reading the letter-number combination written in the square of the checkerboard that was just above that location. These verbal responses were transformed into errors of direction and amplitude that were then analyzed in the same way as movement errors. However, the results obtained were rather noisy, and the conclusions are somewhat tentative.

By and large the line-symmetric approximation seems to be represented explicitly, but the fine tuning implicitly. For example, the variations of visual feedback during practice, which affected the accuracy of fine tuning and thus the errors in open-loop tests, had no reliable effects on the errors of explicit judgements. Sülzenbrück and Heuer (2011) observed a somewhat larger error of explicit judgements after practice with concurrent visual feedback than after practice with terminal feedback; the difference, however, did not approach statistical significance. In contrast, the error observed in the visual open-loop test was reliably larger after practice with concurrent visual feedback than after practice with terminal feedback. Similarly, Sülzenbrück and Heuer (2012) observed a somewhat larger error of explicit judgements after practice with the visible shaft of the lever than after practice with only the cursor being visible, but again the difference failed to reach statistical significance—in contrast to the significant difference between movement errors after the different practice conditions.

In the study of generalization across the workspace, explicit judgements did only marginally deviate from the symmetry approximation (Heuer and Sülzenbrück, 2013b). The fine tuning was essentially absent. This suggests that it was represented implicitly rather than explicitly. However, there was essentially no evidence of fine tuning in the after-effects, which are often used as a measure of implicit adjustments. Nevertheless, the fine tuning was clearly present in open-loop tests in which the presence of the transformation of the sliding lever was instructed. Thus, there is little doubt that the symmetry approximation is explicitly represented, but for the fine tuning the issue is somewhat unsettled. Perhaps it is implicitly represented, but the absence of the lever in the after-effect test served as an effective cue not to invoke the internal model of the tool any more (cf. Kluzik et al., 2008).

### Positions and movement paths

There is some indication that end positions and other characteristics of movements aimed at a target are controlled separately (DiZio and Lackner, 1995; Sainburg and Wang, 2002; Brown et al., 2003). Thus, in principle the internal model of a visuo-motor transformation could map start and target positions on corresponding positions of the hand. Alternatively, of course, it could map desired trajectories of the tip of the lever (or of the cursor). In the first case, only target positions would be transformed, and trajectories would remain those normally found with the particular start-target combinations for the hand. This kind of observation has been reported by Verwey and Heuer (2007) for rapid movements with a non-linear amplitude transformation. Whereas the target positions of the hand were transformed, the velocity profile of the hand was essentially the same as when hand movements to the same targets were produced in the absence of the non-linear amplitude transformation.

In the experiments with the sliding first-order lever we focussed on curvature. Hand movements from a start position to a target have almost straight paths (Morasso, 1981; Abend et al., 1982; Atkeson and Hollerbach, 1985; Kaminski and Gentile, 1986). With the sliding lever, straight paths of the hand result in curved paths of the tip of the lever, as is evident from Figure 1. Almost straight paths of the hand would be expected if only visual target positions were transformed into target positions for the hand. Alternatively, if straight paths of the tip of the lever (and the cursor) were planned and transformed into paths of the hand, these should be appropriately curved.

Transverse movements are known to have a slight concave curvature in general (Wolpert et al., 1994; Haggard and Richardson, 1996; Van Thiel et al., 1998). In two experiments with the sliding first-order lever (Sülzenbrück and Heuer, in preparation) concave curvature of hand movements was observed only in a particular condition. In this condition the kinematic transformation of the lever was present, targets were defined for the tip of the lever, visual feedback was terminal or absent, and the pen was detached from the lever so that there was a constant inertial resistance of the tool rather than the inertial anisotropy of the sliding lever (the dynamic transformation of the lever was absent). When the dynamic transformation of the lever was present, that is, when the pen was attached to the lever, concave curvature of hand movements turned into convex curvature. Whereas concave curvature of transverse hand movements increases the curvature of the cursor paths, convex curvature of the hand movements results in a straightening of the cursor paths. Such an effect of the dynamic transformation has also been observed by Sülzenbrück and Heuer (2010), and it can likely result in straighter paths of the tip of the lever (and the cursor) than of the hand (cf. Heuer and Sülzenbrück, 2009).

When terminal visual feedback is replaced by concurrent visual feedback, curvature of hand paths becomes convex both in the presence and in the absence of the dynamic transformation of the lever. Convex curvature of hand movements is associated with a straightening of the paths of the cursor. This finding on the effects of concurrent visual feedback corresponds to observations made with other types of kinematic transformations when the cursor was visible (Flanagan and Rao, 1995; Wolpert et al., 1995). Thus, processing of visual feedback seems to be critical for straight paths of the cursor. Straight paths of the cursor are thus not based on the internal model of the visuo-motor transformation, but they are a characteristic of visual closed-loop control.

Hand paths change from convex or concave curvature toward straightness when targets are presented for the hand rather than for the tip of the lever, that is, when the kinematic transformation of the sliding lever is absent. This was the case both in the presence (cf. Heuer and Sülzenbrück, 2012a) and in the absence of the dynamic transformation, even when there was no concurrent visual feedback. Nevertheless, the straightening of hand paths under those conditions is likely a consequence of closed-loop control based on proprioceptive rather than visual feedback signals.

Movement execution involves a series of transformations, including the extrinsic transformations implemented by tools. Thus, when a more proximal variable such as the movement of the hand is controlled, the more distal variables such as movements of the effective part of the tool are secondary and result from the transformations. However, control can also refer to a more distal variable such as the trajectory of the effective part of the tool. In this case the more proximal variables are secondary and result from the operations that invert the transformation—the inverse internal model of open-loop control and the closed-loop control based on the sensory registration of the controlled variable. Regarding the question whether control is concerned primarily with more proximal or more distal variables, the distal-control hypothesis has gained weight during the last couple of years as a major ingredient of broader conceptions of action control (Prinz, 1992, 1997; Hommel et al., 2001; Kunde et al., 2004; Kunde, 2006).

The present findings with the sliding lever do not fit the simple distinction between proximal and distal control. Control is distal with respect to movement targets. This must be the case as long as movements serve their purpose, provided that targets are distally defined, that is, for the tip of the lever. But when targets are defined for more proximal variables such as the position of the hand, distal variables can be neglected. Depending on the more distal or more proximal variable for which targets were defined, we found (almost) straight paths of the tip of the lever or of the hand (and correspondingly curved paths of the hand and the tip of the lever, respectively). However, this was true only when concurrent feedback on the path of the tip of the lever or of the hand was available. For the tip of the lever, the only source of concurrent feedback is vision, but for the hand there is proprioception in addition. When targets were defined for the tip of the lever and visual information was no longer available, curvature of the path of the tip of the lever was affected by the dynamic transformation of the tool. Thus, straightness of the path of the tip of the lever seems to result from visual closed-loop control, but not from open-loop control. Consistent with the conclusion of Verwey and Heuer (2007), which was based on findings with a quite different paradigm, the internal model seems to translate only visual targets into hand targets. Thereafter the path of the hand is not a controlled, but an emergent property as long as no closed-loop control of the effective part of the tool is possible. This is different when the targets for the hand are defined directly. In this case proprioceptive feedback is used for closed-loop control.

## Concluding remarks

In this paper we have integrated a number of observations on the mastery of the rather complex transformation of a certain tool, a sliding first-order lever. The study of this tool was motivated both by theoretical and applied considerations. In this section we shall briefly touch upon some open issues from both perspectives.

Learning to operate a sliding first-order lever involves both basic sensori-motor processes and cognitive strategies, likely based on mechanical reasoning to some degree. Thus, there may be differences to less complex extrinsic transformations and to intrinsic transformations. Perhaps these differences are captured by the distinction between cognitive and perceptual learning (Bedford, 1993) or between motor skill acquisition and recalibration (Clower and Boussaoud, 2000). Perhaps a more continuous conception of differences between adjustments to different types of transformations is more appropriate. In any case a theoretical clarification—based on solid experimental data—would be highly desirable. Thus far only fragments of such a theory exist.

In a discussion of differences between learning of intrinsic and extrinsic transformations, Heuer, (1983, p. 36–38) noted two major contrasts. The first one is in terms of the timescales of changes of the transformation (cf. Körding et al., 2007). The other one is in terms of the identity of the object to which discrepant visual and proprioceptive position information refer (cf. Bedford, 1995). As a marker of the type of internal model acquired, after-effects were envisaged which can be observed when the novel transformation is no longer present. After-effects can be conceived as signature of a learned intrinsic transformation, and the absence of after-effects as the signature of a learned extrinsic transformation (cf. Kluzik et al., 2008). A change of the internal model of intrinsic transformations as a result of practice is conceptually similar or even identical to a change of the body schema, a change that also has been inferred from the observation of after-effects (Cardinali et al., 2009).

The distinction between the two kinds of transformation is fuzzy, at least for the learner. He or she is faced with the credit assignment problem whether changes of intrinsic or extrinsic transformations are responsible for the changes of sensori-motor performance. The principles by which the problem is solved are not yet fully clear. There is evidence from prism-adaptation studies for the role of repeated changes between transformations, in the course of which after-effects of the optical displacement disappear (Kravitz, 1972; Welch et al., 1993), and for the role of experienced object identity (Welch, 1972). More recent findings by Kluzik et al. (2008) show reduced after-effects also with the abrupt rather than gradual introduction of a force field, similar to previous observations on extrinsic visuo-motor rotations (Kagerer et al., 1997). Thus, there is likely a gradual transition between characteristics of acquired internal models of extrinsic and intrinsic transformations. Learning of the complex extrinsic transformation of the sliding lever may differ even more from adaptation to intrinsic transformations than learning to use simple tools such as levers and rakes because of the role of mechanical reasoning (Johnson-Frey, 2003).

A valid theoretical framework for adjustments to different types of transformations could also be helpful to structure apparently contradictory results. A particularly conspicuous set of conflicting and opaque findings are those on the effects of concurrent and terminal visual feedback during practice. Even though our own results are largely in line with the trade-off hypothesis according to which better conditions for closed-loop control during practice result in poorer acquisition of an internal model of the transformation, findings from other laboratories strongly suggest the existence of not yet identified conditions that critically modulate the effect of practice conditions (cf. Sülzenbrück, 2012).

The theoretical framework at stake would certainly have to build on a distinction of different processes involved in mastering complex visuo-motor transformations. In this paper we have not only distinguished between implicit and explicit adjustments, but also between a discrete approximation, that is rapidly acquired, and a slowly acquired graded fine tuning. Similar distinctions between discrete approximations and graded fine tuning have been suggested by Abeele and Bock (2001) and Werner and Bock (2010). In addition, formal models with two or more concurrent processes operating at different rates have been proposed to account for a large set of findings (Smith et al., 2006; Lee and Schweighofer, 2009). At present the relations between the different two- or multi-process models are not clear.

Turning to the applied perspective, the sliding first-order lever shares fundamental mechanical characteristics with the tools used in minimal access surgery. To the extent that surgical-skills training becomes separated from the operating theater and physical or virtual simulators are added to the traditional apprenticeship model of surgical training, principles of motor learning and performance gain relevance for the design of training devices and procedures (e.g., Wulf et al., 2010). Of course, the generalization of basic-research findings to the design of training procedures needs specific validations. Nevertheless, the findings reported in this paper suggest a few practical considerations.

According to the trade-off hypothesis of closed-loop control during practice and the acquisition of an internal model, visual feedback during (simulator) practice should be poor so that a more accurate internal model of the transformation of the tool can be developed. In contrast, when performance rather than learning is critical, conditions for visual feedback should be optimized, e.g., by using a large visual field to the extent that this is possible. Even with an optimized internal model, performance—in particular with respect to accuracy—will continue to depend critically on visual closed-loop control. Finally, training should take the specificity of the internal model for certain regions of the workspace of the tool into account. Therefore, it should cover the whole workspace and not only parts of it.

### Conflict of interest statement

The authors declare that the research was conducted in the absence of any commercial or financial relationships that could be construed as a potential conflict of interest.
